# Microfluidic lung-on-a-chip for evaluating inflammation and toxicity from environmental pollutants

**DOI:** 10.3389/fbioe.2026.1942916

**Published:** 2026-09-23

**Authors:** Fen Zhang, Haiyang Jia, Fei Yu, Yingjie Bao, Dan Wang

**Affiliations:** 1 School of Food and Bioengineering, Xuzhou University of Technology, Xuzhou, China; 2 School of Physics and New Energy, Xuzhou University of Technology, Xuzhou, China

**Keywords:** environmental pollutant, inflammation, lung-on-a-chip, microfluidics, toxicity

## Abstract

Environmental pollutants pose an escalating health threat, yet traditional toxicity models are inherently limited in recapitulating complex human physiology. As an emerging organ-on-a-chip technology, microfluidic lung-on-a-chip employs microengineering approaches to precisely replicate the key structural features and dynamic physiological microenvironment of the human lung *in vitro*, thereby providing a highly biomimetic research platform for toxicity evaluation of environmental pollutants. This review aims to summarize the core principles of this technology, its current application status in modelling pollutant exposure and toxic responses, and to analyze its advantages including biomimetic modeling and real-time monitoring over conventional *in vitro* models, as well as the challenges currently faced involving chip standardization, operational complexity, and subsystem integration. The article focuses on reviewing the latest research progress of microfluidic lung on a chip in assessing the inflammation and toxicity of atmospheric particulate matter and pathogen, volatile organic compounds and aromatic hydrocarbon, and emerging micro/nanomaterials, and further envisions its broad application prospects in advancing studies in environmental toxicology, precision risk assessment, and personalized health prediction.

## 1 Introduction

Environmental pollutants pose a global public health threat, with the respiratory system as a primary target of exposure. Pollutants such as fine particulate matter (PM), which can penetrate deep into the alveoli and induce oxidative stress, significantly increase the risk of respiratory and cardiovascular diseases (Wang et al., 2025). Microplastics and nanoplastics have emerged as ubiquitous environmental pollutants in recent years, with their detection frequently documented across a range of environmental media (Winiarska et al., 2024). These particles can enter the human body via inhalation, thereby exacerbating the health risks (Uchmanowicz et al., 2025). Such pollutants including PM_2.5_, ozone, nanomaterials, and various chemical substances can elicit a range of toxic effects in lung tissue, such as inflammation, oxidative stress, and barrier dysfunction (Guerra-Flórez et al., 2023). However, conventional toxicity assessment models, including animal experiments and static two-dimensional (2D) cell cultures, suffer from significant limitations. Animal models exhibit species differences that preclude accurate prediction of human physiological responses, while static 2D cell cultures fail to recapitulate the complex three-dimensional (3D) tissue architecture, cell–cell interactions, and critical physiological microenvironment (e.g., fluid condition, air–liquid interface, and respiration-related mechanical strain) present *in vivo*, resulting in low throughput and poor clinical relevance (Sun et al., 2021; Irrechukwu et al., 2023).

To overcome these challenges, microfluidic lung-on-a-chip technology has emerged (Huh et al., 2010; Ringquist et al., 2026). This technology integrates microfluidics, cell biology, and tissue engineering, enabling precise control over hydrodynamics, mechanical forces (e.g., cyclic stretching that mimics respiration), and co-culture of multiple cell types on a microscale chip, thereby constructing 3D lung tissue models with high physiological relevance (Wu et al., 2025). For instance, a lung-on-a-chip based on a biomimetic hydrogel membrane better recapitulates the composition and mechanical properties of the alveolar basement membrane and successfully reconstructs the alveolar–capillary barrier function (Shen et al., 2023). This platform offers an unprecedented tool for understanding the interactions between pollutants and lung tissue under dynamic conditions. In recent years, microfluidic lung-on-a-chip devices (Table 1) have been widely employed to simulate inhalation exposure to various environmental pollutants and to investigate their toxicity pathways. Studies have demonstrated that this platform can effectively assess acrolein-induced disruption of the alveolar–capillary barrier, increased release of pro-inflammatory cytokines, and mitochondrial dysfunction (Xu et al., 2026). With respect to PM, lung-on-a-chip models have revealed that PM_10_ exposure can significantly elevate pathological biomarkers such as IL-6 and IL-13, recapitulating pathological conditions like asthma and chronic obstructive pulmonary disease (Jabbar et al., 2022). Of particular note, this technology has shown great potential in elucidating the pulmonary toxicity mechanisms of emerging pollutants such as nanoplastics. Research indicates that, compared with micro-/nanoplastics derived from polypropylene or polyethylene terephthalate, polystyrene particles cause the most severe impairment to the biophysical function of pulmonary surfactant; the heterogeneous aggregates they form further exacerbate lung injury at the alveolar–capillary interface (Xu et al., 2023). Collectively, these application cases fully demonstrate the efficacy and importance of microfluidic lung-on-a-chip in toxicity assessment of environmental pollutants.

**TABLE 1:** Summarization of recent lung-on-a-chip for environmental assessment.

Study objectives (Refs)	Lung model (cell source)	Chip materials and fabrication	Pollutant (dosage)	Endpoints (readouts)	Advantage	Limitation
PM_10_-induced pulmonary influence investigation (Jabbar et al., 2022)	Airway model (Human small airway epithelial cells)	Glass/MED-6033 silicon elastomer 3D printing	PM_10_ (7.5–151 μg/mL)	Epithelium integrity (impedance) Oxidative stress (ROS) Inflammation (IL-6, IL-13, TNF-α)	Real-time analysis; sensor integration; fluorescence tracking	Short-term simulation; insufficient flux; difficult standardization
Assessing PM_2.5_-induced toxicity (Guan et al., 2021)	Alveolar–capillary barrier (A549 cells)	PDMS Soft lithography	PM_2.5_ (100 μg/mL)	Cytotoxicity (dead cells) Oxidative stress (ROS) Inflammation (IL-1α, IL-6, IFN-α)	Real-time analysis; barrier integrity	Short-term simulation; insufficient flux; difficult standardization
Assessing PM_0.5_ pollution effect under breathing mechanostress (Kaya and Yesil-Celiktas, 2024)	Lung epithelial cell layer (Calu-3 cells)	PDMS Soft lithography	PM_0.5_ (800 μg/mL)	Cytotoxicity (WST-1 assay) Inflammation (IFN-α)	Real-time analysis	Low and short-term simulation; insufficient flux; difficult standardization
Modelling the human severe influenza infection response (Ringquist et al., 2026)	Small airway model (Primary small airway epithelial cells, umbilical vein endothelial cells, lung fibroblasts, macrophages)	PDMS Soft lithography	H1N1 (an multiplicity of infection of 10)	Epithelial damage (LDH) Inflammation (IL-10, IL-1β, GM-CSF, CXCL10, IFN-λ1, IP-10, TNF-α)	Real-time analysis; high simulation	Short-term simulation; insufficient flux; difficult standardization
Assessing cytokine secretion triggered by VOCs (Goldman-Pinkovich et al., 2026)	Bronchial airway model (human induced pluripotent stem cells)	PDMS Soft lithography	Benzene (0.01 µM and 0.1 mM)	Cytotoxicity (LDH) Inflammation (IL-10, IL-1β)	Real-time analysis; high simulation	Short-term simulation; insufficient flux; difficult standardization
Investigation of pollutant-induced lung inflammation and injury (Zhang et al., 2020)	Alveolar–capillary barrier (Human pulmonary alveolar epithelial cells and pulmonary microvascular endothelial cells)	PDMS Soft lithography	Benzopyrene (5–500 µM)	Barrier integrity (F-actin) Cytotoxicity (dead cells) Oxidative stress (ROS) Inflammation (IL-6, IL-8, MCP-1, IP-10, GM-CSF)	Real-time analysis; high simulation	Short-term simulation; insufficient flux; difficult standardization
Quantification of nanoplastics-induced oxidative stress (Zhao et al., 2024)	Lung epithelial cell layer (A549 cells)	PDMS Soft lithography	Polystyrene-nanoparticles (50 μg/mL)	Cytotoxicity (dead cells) Oxidative stress (ROS)	Periodic respiratory simulation	Short-term simulation; insufficient flux; difficult standardization
Assessing zinc oxide nanoparticles induced cellular response (Arathi et al., 2022)	Lung epithelial cell layer (A549 cells)	PDMS Soft lithography	Zinc oxide nanoparticles (10–320 μg/mL)	Epithelium integrity (F-actin) Cytotoxicity (dead cells)	Real-time analysis	Low and short-term simulation; insufficient flux; difficult standardization
Investigation of nanoplastic-triggered lung inflammation and injury dynamics (Zhang et al., 2025)	Alveolar–capillary barrier; gradient control (Human pulmonary alveolar epithelial cells and pulmonary microvascular endothelial cells)	PDMS Soft lithography	Polystyrene-nanoparticles (17–100 μg/mL)	Barrier integrity (F-actin) Cytotoxicity (dead cells) Oxidative stress (ROS) Inflammation (IL-6, IL-8, MCP-1, IP-10, GM-CSF, RANTES)	Efficient gradient generation; high simulation real-time analysis; multi-parallel evaluation	Short-term simulation; difficult standardization

The lung-on-a-chip systems have been reviewed (Singh et al., 2021; Sun et al., 2021; Wang et al., 2023) from various perspectives including smoking-related toxicology, atmospheric pollutant exposure assessment, pathological modeling and drug discovery. The organ-on-a-chip platforms for micro/nanoplastic toxicology has been reviewed too (Abdessalam et al., 2025). It is worth noting that there are few reviews on the lung-on-a-chip for model construction and hazard evaluation of various environmental pollutants with emphasis on recent advancements, especially the past 3 years. Here, we overview recent advances in lung-on-a-chip technology for assessing inflammation and toxicity induced by diverse environmental pollutants including PM and pathogen, volatile organic compounds (VOCs) and aromatic hydrocarbon, and micro/nanomaterials. Representative cases of recent applications are introduced and discussed. Technical advantages, challenges, and future application prospects of the technology are also highlighted. Finally, we conclude with a discussion of the development of lung-on-a-chip for environmental assessment.

## 2 Technical principles and model construction of lung-on-a-chip

### 2.1 Key components and chip design

The key components of lung-on-a-chip are designed to precisely recapitulate the complex physiological microenvironment of the human lung. The chip architecture generally comprises interconnected microchannels/chambers and a porous membrane (Figure 1A) that separates an air chamber from a liquid chamber, thereby reconstituting the alveolar–capillary barrier, being a key physiological structure *in vitro* (Huh et al., 2010). These chips are typically fabricated from materials with good biocompatibility and optical transparency, such as polydimethylsiloxane (PDMS), using soft lithography (Yang et al., 2020). Compared with other chip materials like glass and polymethyl methacrylate, PDMS is widely used in lung-on-a-chip fabrication, owing to its biocompatibility, gas permeability, optical transparency, chemical inertness, molding convenience, and cost-effectiveness. The porous polycarbonate membrane, polycaprolactone and collagen nanofiber, and Matrigel are typically employed for constructing the biomimetic basement membrane (Zhang et al., 2020; Kanabekova et al., 2024). To more faithfully mimic the sac-like morphology of alveoli, some studies have developed a soap-bubble-inspired vesicular hydrogel membrane, which can stably withstand respiration-like cyclic mechanical stretching and fluid flow, providing a critical platform for constructing a more biomimetic alveolar–capillary barrier (She et al., 2024). In terms of mechanical and hydrodynamic control, micropumps and precision pressure control systems are employed to accurately simulate respiration-like cyclic mechanical strain (typically 10% at 0.2 Hz in human alveoli) and capillary blood flow profile (e.g., 1 mm/s). As a representative condition, a flow rate of less than 1 μL/min was chosen to apply a physiologically relevant fluid shear stress (∼1 dyne/cm^2^) for biomimetic reconstruction (Huh et al., 2012; Li et al., 2024; Zhang et al., 2020). These physical stimuli are essential for maintaining the normal phenotype and function of alveolar epithelial cells and endothelial cells (Huh et al., 2010; Koornneef et al., 2025). Cell co-culture systems form the basis for model functionalization, typically involving co-culture of human-derived alveolar epithelial cells (e.g., A549 cells, primary cells, and induced pluripotent stem cell-derived cells) with human vascular endothelial cells at an air–liquid interface (Kim et al., 2023). To construct more complex microphysiological systems, researchers have also introduced immune cells (e.g., macrophages) and fibroblasts into the models to mimic key cell–cell interactions and immune responses within the pulmonary microenvironment (Chen et al., 2023). In the pulmonary microenvironment, the alveolar epithelial cells closely interact with resident alveolar macrophages. Airborne pollutant exposure disrupts pulmonary immune homeostasis via oxidative stress-driven activation of epithelial–macrophage crosstalk (Dubey et al., 2026). Alarmins released from injured epithelia promote M1 macrophage polarization through NF-κB/NLRP3 signaling, releasing pro-inflammatory cytokines that recruit neutrophils and damage the epithelial barrier. Persistent imbalance between M1 and M2 macrophage responses culminates in chronic inflammation and fibrosis. Lung-on-a-chip platforms, incorporating cyclic stretch and air–liquid interface, enable physiologically relevant investigation of these dynamic immunotoxicological mechanisms. Typically, an aerosol delivery system is applied for on-chip pollutant exposure. The system mainly consists of a pollutant storage container and either a vacuum-operated inhalation simulator or a stepper-motor-driven microrespirator (Benam et al., 2016; Xue et al., 2023). The outlet of the chosen device is connected to the inlet of the chip for the pollutant introduction.

**FIGURE 1:**
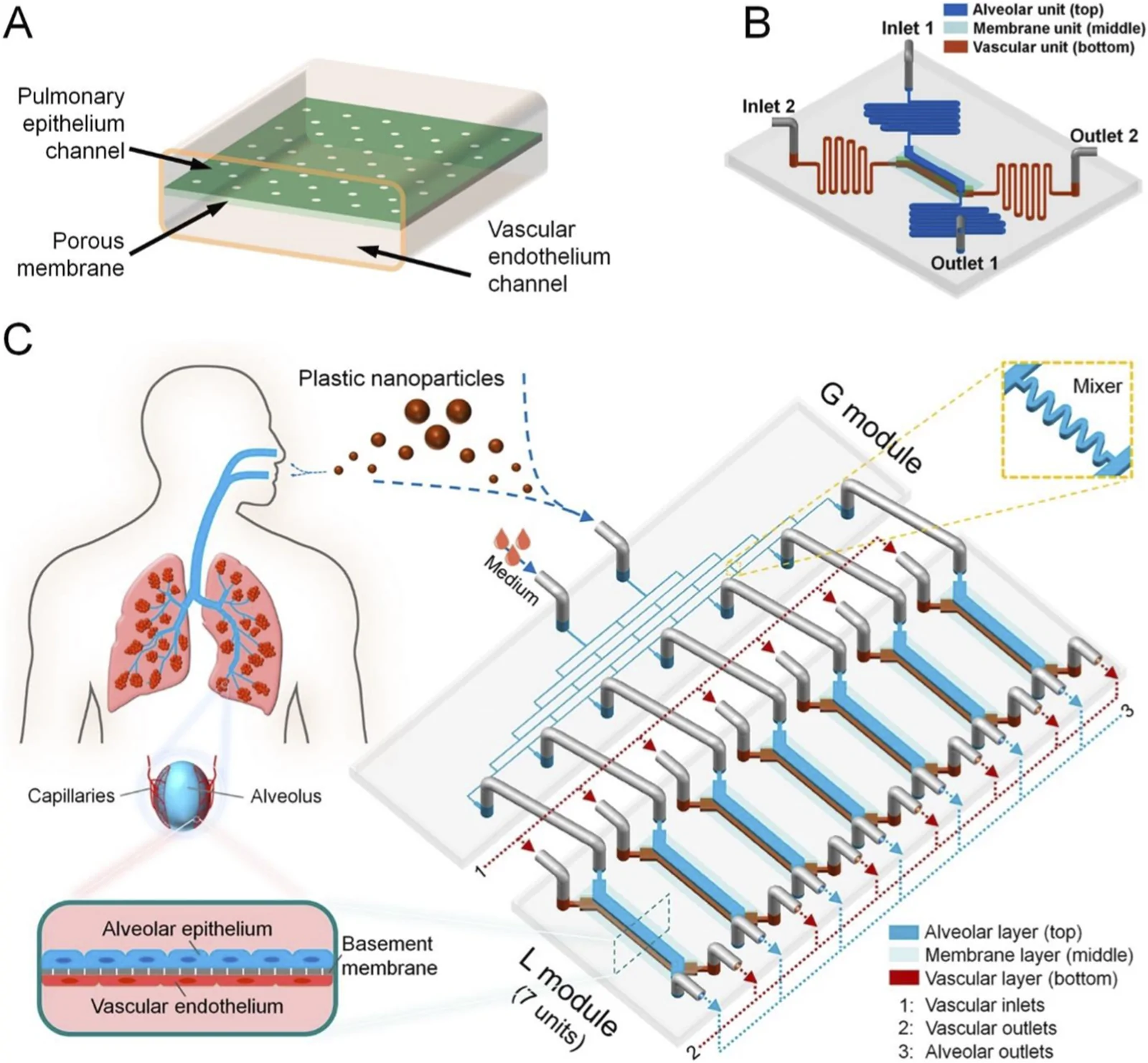
Lung-on-a-chip systems for environmental assessment. **(A)** Schematic diagram of the typical lung chip consisting of a pulmonary epithelium channel, a vascular endothelium channel, and a porous membrane interposed between them. **(B)** Microfluidic human pulmonary alveolus system for investigating benzopyrene-induced lung inflammation and injury. Reproduced with permission (Zhang et al., 2020). Copyright 2020, American Chemical Society. **(C)** Integrated microfluidic lung chip for assessing nanoplastic-triggered lung inflammation and toxicity. Reproduced with permission (Zhang et al., 2025). Copyright 2025, Elsevier.

### 2.2 Model functionalization and validation

Functionalization of the lung-on-a-chip model must be validated through a series of quantitative endpoints to ensure its reliability in mimicking pulmonary physiological and pathological processes. Assessment of barrier function is central to validating alveolar–capillary integrity, typically accomplished by measuring transepithelial electrical resistance and performing fluorescent tracer permeability assays (Meghani et al., 2020). These methods enable quantitative evaluation of changes in barrier permeability following exposure to environmental pollutants (e.g., PM), thereby reflecting early structural damage to lung tissue induced by such pollutants (Koornneef et al., 2025). For the simulation of metabolic and clearance functions, advanced lung-on-a-chip models aim to incorporate metabolic activities such as cytochrome P450 enzymes to assess the biotransformation of pollutants within the lung (Yang et al., 2021). Meanwhile, by modelling mucociliary clearance, the deposition and clearance kinetics of airborne particles can be investigated (Yang et al., 2020). To achieve multi-parameter dynamic monitoring, lung-on-a-chip devices are often coupled with online or offline high-sensitivity detection systems. For instance, integration of real-time fluorescence microscopy, enzyme-linked immunosorbent assay, and mass spectrometry allows continuous monitoring of cell viability, secretion of inflammatory cytokines (e.g., IL-6, IL-8), oxidative stress markers, and genomic/proteomic alterations following pollutant exposure (Chen et al., 2023; Liu et al., 2024). In addition, some studies have integrated flexible inkjet-printed pH sensors into the lung-on-a-chip for real-time monitoring of pH fluctuations in the simulated alveolar microenvironment, thereby providing a new tool for studying pollutant-induced chemical changes in the local milieu (Boček et al., 2026). Collectively, these comprehensive validation endpoints ensure that the lung-on-a-chip model possesses high physiological relevance and predictive value in environmental toxicology research.

## 3 Application of lung-on-a-chip in environmental assessment

### 3.1 Toxicological mechanisms of atmospheric PM and pathogen

Microfluidic lung-on-a-chip provides an unprecedentedly precise platform for investigating the toxicological mechanisms of atmospheric PM and pathogen (Wang et al., 2023). Through finely engineered airflow channels, researchers can accurately control the flow of aerosols containing PM_2.5_ or pathogen (e.g., bacteria and viruses), simulating their deposition patterns in the alveolar region (Amin Arefi et al., 2020; Li et al., 2024; Sengupta et al., 2025; Guan et al., 2021). This dynamic exposure model overcomes the limitations of conventional static cultures and enables quantitative evaluation of the deposition efficiency of particles with different sizes and chemical compositions onto alveolar epithelial cells and resident macrophages (Xu et al., 2020; Kaya and Yesil-Celiktas, 2024). Physiological breathing patterns simulated within the chip such as high-flow conditions during exercise or the “inhalation-hold-exhalation” pattern of smokers significantly increase particle deposition.

In terms of elucidating early molecular events triggered by PM or pathogen, the lung-on-a-chip demonstrates remarkable capability. For example, Ringquist et al. recently developed a human lung-on-a-chip model (Ringquist et al., 2026) that recapitulates immune responses to severe H1N1 infection, including cytokine storm and epithelial damage. Mechanistically, it revealed that IL-1β and TNF-α exert opposing roles in initiating and regulating the cytokine storm, and identified the stromal–immune CXCL12–CXCR4 axis as a critical pathway governing immune activation during infection. This work underscore the essential interplay between stromal and immune cells in driving severe lung pathology. For PM-related assessment, a microfluidic lung chip was developed (Guan et al., 2021) to simulate the respiratory microenvironment at the alveolar-capillary interface for determining the mechanism of PM_2.5_ toxicity. The results demonstrated that exposure to PM_2.5_ at a concentration of 100 μg/mL induces severe oxidative stress and may ultimately lead to cell death. Another study utilizing a microfluidic co-culture system based on the alveolar–capillary interface dynamically monitored the temporal changes in pro-inflammatory cytokine release (e.g., IL-6 and TNF-α) from epithelial and endothelial cells following PM_10_ exposure (Jabbar et al., 2022). Concurrently, the system successfully captured oxidative stress responses such as reactive oxygen species (ROS) generation, thereby clarifying the early pathways leading to acute lung injury and chronic diseases induced by PM. Furthermore, lung-on-a-chip models effectively assess the disruption of alveolar–capillary barrier function caused by PM exposure. Research has confirmed that PM_2.5_ exposure leads to aberrant expression and distribution of tight junction proteins, accompanied by a decline in transepithelial electrical resistance and increased barrier permeability (Koornneef et al., 2025; Xu et al., 2020). This barrier disruption mimics the pathological process by which PM facilitates the translocation of allergens or pathogens, providing direct evidence for understanding its pathogenic mechanisms. For the underlying toxicological mechanism, PM exposure increases ROS production, activates endoplasmic reticulum stress, and upregulates the PERK-eIF2α signaling pathway, which in turn promotes elevated expression of the downstream pro-apoptotic proteins, which cooperate with activated Caspase-3 to induce apoptosis of lung cells, causing the lung tissue damage and dysfunction (Guan et al., 2021; Wang et al., 2023). In addition, a recent lung-on-a-chip study demonstrated that cyclic stretching promotes macrophage-mediated PM_2.5_ translocation across the air-blood barrier by both enhancing dTHP-1 phagocytosis and transmigration, and disrupting epithelial tight junctions (Li et al., 2026). For chip operation, the reported lung platforms commonly adopted the single group mode for studying the pollutant-induced lung pathology. Multi-parallel group mode and multi-functional integration for synchronous and large-scale exploration of dose-gradient environmental assessment in the single chip remains largely out of reach.

### 3.2 Toxicity assessment of VOCs and aromatic hydrocarbon

Microfluidic lung-on-a-chip, through its integrated gas or liquid perfusion system, enables the construction of highly biomimetic dynamic exposure models for VOCs and aromatic hydrocarbon. This system can simulate the periodic inhalation exposure to VOCs (e.g., benzene, formaldehyde) during human respiration, thus overcoming the drawbacks of traditional static culture dishes, where exposure concentrations are inaccurate and fail to reflect actual inhalation dynamics (Singh et al., 2021; Goldman-Pinkovich et al., 2026). This dynamic exposure capability is particularly critical for studying pollutants with metabolic activity. Using lung cell models cultured on-chip that possess metabolic enzyme activities (e.g., CYP2E1), researchers can delve into the DNA adduct damage and apoptosis induced by reactive intermediates generated from pulmonary metabolism of VOCs (Rothbauer et al., 2021). Further, a microfluidic pulmonary alveolus system (Zhang et al., 2020) facilitates convenient comparison of the differential sensitivity of various cell types (e.g., epithelial vs. endothelial cells) to aromatic hydrocarbon toxicity, revealing cell-specific injury mechanisms (Figure 1B). Benzopyrene stimulation at a dose of over 20 µM for 2 days was proved to the structural integrity of alveolar-capillary interfaces, as well as the production of various inflammatory cytokines like IL-6, IL-8, and MCP-1. The endothelium in the interface was more chemosensitive and exhibited stronger activity of ROS generation than the epithelium in the chip. To more realistically reflect complex co-exposure scenarios involving multiple pollutants in the actual environment, researchers have designed multi-channel microfluidic chips. These chips can be used for simultaneous or sequential exposure to combined pollutants such as PM and VOCs, enabling the study of synergistic or antagonistic toxic effects (Abdessalam et al., 2025). Mechanically, exposure to these chemical pollutants in the lung-on-a-chip model directly injure the alveolar epithelium via ROS-induced oxidative stress and activation of AhR/NF-κB signaling (Goldman-Pinkovich et al., 2026; Yin et al., 2025; Wang et al., 2023), leading to barrier disruption and pro-inflammatory cytokine release, which further trigger endoplasmic reticulum stress, apoptosis, and immune cell infiltration, while disturbing stromal-epithelial crosstalk, which could ultimately result in chronic inflammation, fibrotic remodeling, or persistent pulmonary dysfunction.

### 3.3 Safety evaluation of micro/nanomaterials

Microfluidic lung-on-a-chip exhibits substantial potential in evaluating the pulmonary toxicity of micro/nanomaterials (Zhang et al., 2018). Under simulated physiological fluid conditions, this platform allows the investigation of the agglomeration, sedimentation, and formation of “protein coronas” with biomolecules for nanomaterials such as carbon nanotubes and zinc oxide nanoparticles within the chip (Rabbani et al., 2020; Hao et al., 2019). More importantly, chip technology can directly link these physicochemical behaviors of nanomaterials to their inflammatory and cytotoxic effects (Yang et al., 2023). For example, studies can observe the intracellular localization of nanomaterials after cellular uptake and the consequent subcellular events, including ROS generation, mitochondrial damage, or apoptosis (Prasad et al., 2021). Zhang and coworkers developed a microfluidic lung-on-a-chip system (Zhang et al., 2025) integrated with a nanoparticle concentration gradient generator for dynamic, dose-dependent assessment of inflammatory and injury responses elicited by polystyrene nanoparticles on a reconstructed human alveolar barrier (Figure 1C). They found that nanoplastic internalization by alveolar epithelial cells positively correlated with the applied nanoparticle doses and also exhibited a duration-dependent increase, thereby promoting disintegration of the alveolar-capillary interface, cellular oxidative stress, proinflammatory protein secretion, and apoptosis. The results demonstrated that low-dose nanoplastics elicit defensive alveolar responses, whereas high-dose exposure (≥50 μg/mL) induces severe inflammatory and cytotoxic effects involving oxidative stress imbalance, alveolar–capillary barrier disruption, proinflammatory cytokine release, and apoptosis. These findings underscore the potential health risks of NPs and highlight the urgent need for environmental surveillance and protective measures. This work provides a proof-of-concept prototype for pulmonary toxicity assessment of nanoplastics. Unlike previous lung chips above, this system combines automated gradient generation, multi-dose co-culture testing, and parallel organ-response profiling. Another study compared the toxicity of L-cysteine-coated zinc oxide nanoparticles to alveolar epithelial cells (A549) under traditional culture versus microfluidic lung-on-a-chip conditions, revealing reduced cell death in the chip environment, suggesting that microenvironmental factors such as hydrodynamics significantly influence nanotoxicity (Arathi et al., 2022). For the underlying toxicological mechanism, micro/nanomaterials can be internalized by alveolar epithelial cells and macrophages, triggering excessive ROS production and oxidative stress, which subsequently activate pro-inflammatory NF-κB and MAPK signaling pathways, leading to cytokine release and inflammatory cell infiltration (Guo et al., 2026; Zhang et al., 2025). These materials may also disrupt mitochondrial function, induce endoplasmic reticulum stress, and activate the unfolded protein response, ultimately promoting apoptosis, pyroptosis, or autophagy-dependent cell death, while impairing the alveolar-capillary barrier and promoting fibrotic remodeling. For the inhalation toxicity of airborne nanoplastics, the lung-on-a-chip provides an advanced tool for preliminary exploration by simulating the inhalation process, which support investigating the mechanical damage to epithelial cells caused by physical irritation and chronic inflammatory responses, thereby filling a gap in in vitro models in this field (Huh et al., 2010). Lung-on-a-chip experiments demonstrated that cyclic stretching significantly enhances the cellular uptake of nanoplastics mainly via caveolin-mediated endocytosis, thereby exacerbating both inflammation (e.g., ROS generation) and cytotoxicity (i.e., cell death) (Zhao et al., 2024). However, these chips was typically on based on the short-term culture. The development of the lung-on-a-chip system that enables long-term culture and pollutant stimulation is necessary for research on chronic lung pathogenesis.

## 4 Technical advantages, challenges, and future application prospects

### 4.1 Advantages and challenges of microfluidic lung-on-a-chip

Microfluidic lung-on-a-chip offers substantial advantages over conventional 2D cultures and animal models. Its primary strength is high physiological relevance: it recapitulates the epithelium and endothelium layer arrangement in 3D (i.e., lung tissue interfaces), mechanical forces (e.g., cyclic stretch), and dynamic fluidics, while supporting co-culture of epithelial, endothelial, and fibroblast cells, thus faithfully mimicking the lung microenvironment (Choi et al., 2025). Although not all lung-on-a-chip systems reported to date can achieve this level of physiological recapitulation. For instance, human pluripotent stem cell-derived lung models exposed to PM exhibit xenobiotic metabolism and oxidative stress responses that align better with *in vivo* physiology than static 2D cultures, yielding more predictive toxicity data (Zhou et al., 2024). Additionally, the chip enables real-time, non-invasive monitoring via integrated imaging, capturing early transient events like ROS bursts. Its small footprint permits microscale manipulation, reducing reagent and cell usage, and aligns with the 3R principles, offering a human-relevant alternative to animal testing (Low et al., 2020; Wang et al., 2026). These features are largely distinct from those of recently reported bioreactor system for the study of pollutant impact on the single lung epithelium, which has demonstrated excellent performance in cyclic strain, basal perfusion, and aerosol delivery (Cacopardo et al., 2026). Furthermore, several limitations and challenges hinder the widespread application of the lung-on-a-chip technology. Foremost is the lack of standardization: variations in chip design, cell sources, culture conditions, and exposure parameters across labs compromise data comparability and cross-validation (Yin et al., 2025). Complexity and high costs, requiring interdisciplinary expertise and expensive equipment, also limit accessibility (Sakthivel et al., 2026). Moreover, long-term culture remains a bottleneck; most models sustain function for only days to weeks, insufficient for chronic low-dose effects (e.g., cadmium-induced fibrosis or carcinogenesis), which demand extended exposure periods (Huang et al., 2023). Importantly, subsystem integration of the lung chip is critical for multifunctional manipulation and analysis in large-scale. Overcoming these obstacles requires interdisciplinary collaboration and technological innovation.

### 4.2 Future application prospects and development

Lung-on-a-chip technology holds promise for toxicology, but several critical gaps impede its widespread application. For personalized precision toxicology, stem cell-derived lung cells often exhibit immature phenotypes and poor metabolic competence, which undermines their predictive accuracy. Standardization of differentiation protocols and quality-control assays (e.g., TEER, surfactant proteins) is urgently needed. Long-term culture is also hindered by evaporation and material adsorption. These issues require engineering fixes such as active humidification and low-adsorption coatings. For multi-organ integration, the main challenge is not mere connection but preserving organ-specific microenvironments and correct size ratios. Most current designs use a single shared medium, which cannot replicate physiological metabolite gradients. Modular, independently perfused units with semi-permeable interfaces are preferable, yet standardized connectors and operating protocols are still lacking (Sakthivel et al., 2026). For artificial intelligence-driven risk assessment, low throughput and inconsistent data formats limit training datasets. Instead of merely generating more data, we need open-access repositories with standardized metadata and active learning to prioritize informative experiments. Automated image analysis can help convert multi-omics into machine-readable features. A tiered validation strategy, starting with reference chemicals and progressing to mixtures, with iterative feedback between models and chip experiments, could eventually establish regulatory acceptance (Lang et al., 2025; Wang et al., 2026). This would offer human-based evidence to support safety thresholds and regulation of diverse environmental pollutants, marking a major shift in toxicology.

## 5 Conclusion

Microfluidic lung-on-a-chip can recapitulate the tissue interfaces, mechanical forces, and dynamic microenvironment of the human lung. As reviewed, it has been successfully applied to assess toxicity of atmospheric PM and pathogen, VOCs and aromatic hydrocarbon, and micro/nanomaterials. Its principal advantage over static cultures and animal models lies in real-time, dynamic monitoring of multicellular interactions at the air–liquid interface, revealing inflammatory cascades, barrier disruption, and aberrant signaling—shifting toxicology toward mechanistic insight. Currently, the technology is transitioning from proof-of-concept to standardization, yet faces hurdles: lack of uniform protocols for chip design, cell sources, and endpoints compromises reproducibility; long-term culture and cost remain bottlenecks for chronic exposure studies and high-throughput applications. Nonetheless, its physiological relevance underpins predictive value, and these limitations are phase-specific challenges, not fundamental flaws. Future directions include personalization via patient-derived cells for precision risk assessment, and systemic expansion through multi-organ interconnection (e.g., lung-liver) to model complex exposures. Ultimately, lung-on-a-chip represents a paradigm shift, bridging *in vitro* and human physiology. With standardization and artificial intelligence integration, it will support evidence-based quality standards, priority pollutant identification, and personalized interventions for environmental health.
